# Supplementary material for: Characterization of exhaled e-cigarette aerosols in a vape shop using a field-portable holographic on-chip microscope
Source: Sci Rep. 2022 Feb 24;12:3175. doi: 10.1038/s41598-022-07150-2 (PMC8873257; doi:10.1038/s41598-022-07150-2)
Supplement: Supplementary file 5 — Supplementary Information. [file 41598_2022_7150_MOESM5_ESM.pdf]

Characterization of exhaled e-cigarette aerosols in a vape shop using a field-portable holographic on-chip microscope

Ege Çetintaş[1,2,3] email: egecetintas1@g.ucla.edu

Yi Luo[1,2,3] email: yluo2016@ucla.edu

Charlene Nguyen[4] email: charchar626@gmail.com

Yuening Guo[4] email: ynguo94@g.ucla.edu

Liqiao Li[4] email: liqiao93@g.ucla.edu

Yifang Zhu[4] email: yifang@ucla.edu

Aydogan Ozcan[1,2,3,5,*] email: ozcan@ucla.edu

[1]Electrical and Computer Engineering Department, University of California, Los Angeles, California 90095, USA

[2]Bioengineering Department, University of California, Los Angeles, California 90095, USA

[3]California Nano Systems Institute (CNSI), University of California, Los Angeles, California 90095, USA

[4]Department of Environmental Health Sciences, University of California, Los Angeles, California 90095, USA

[5]David Geffen School of Medicine, University of California, Los Angeles, California 90095, USA

[*]Correspondence: Prof. Aydogan Ozcan

E-mail: ozcan@ucla.edu

Address: 420 Westwood Plaza, Engr. IV 68-119, UCLA, Los Angeles, CA 90095, USA

Tel: +1(310)825-0915

Fax: +1(310)206-4685

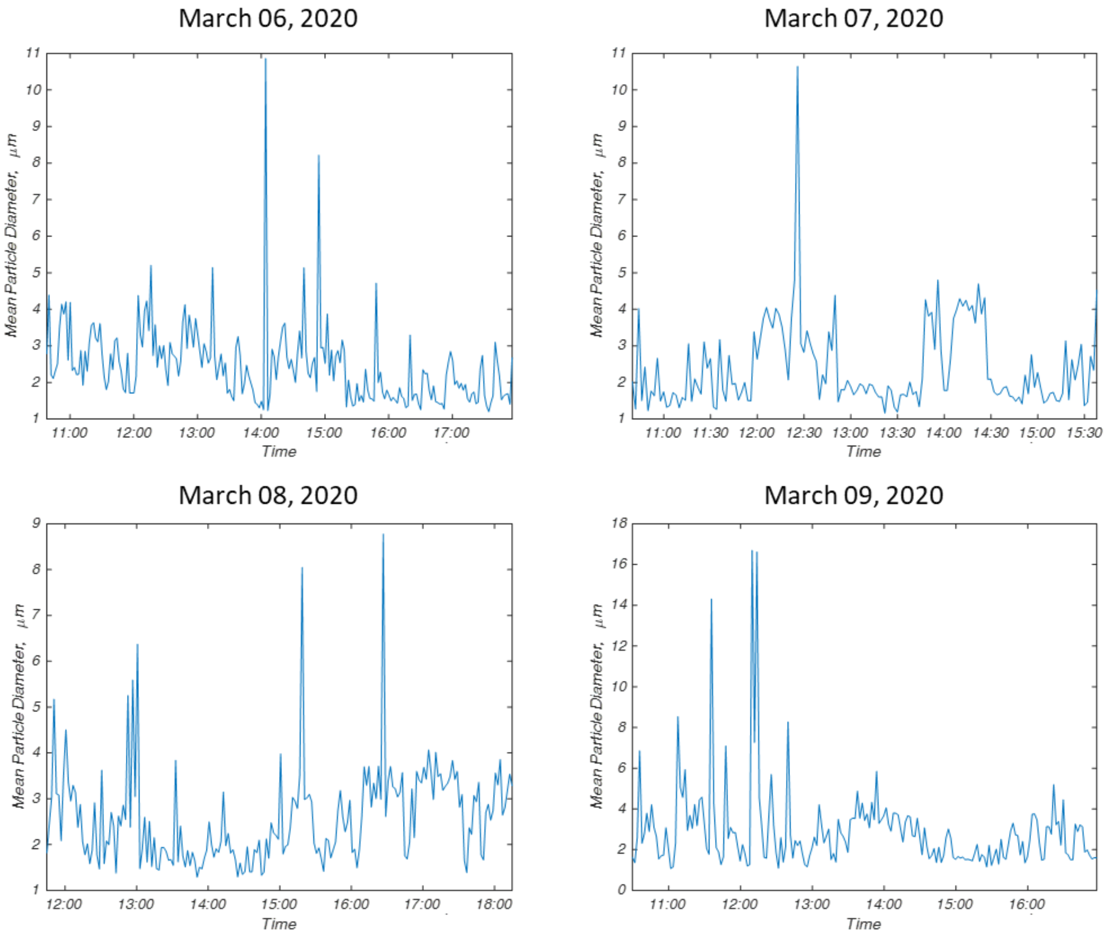

Supplementary Figure S1. The change of the mean particle diameter measured by the APS system throughout different days of the field experiments. The particle sizing resolution of the APS device is under 500 nm. The variations shown in these plots reflect the complexity and rapid changes of the indoor environment dynamics.

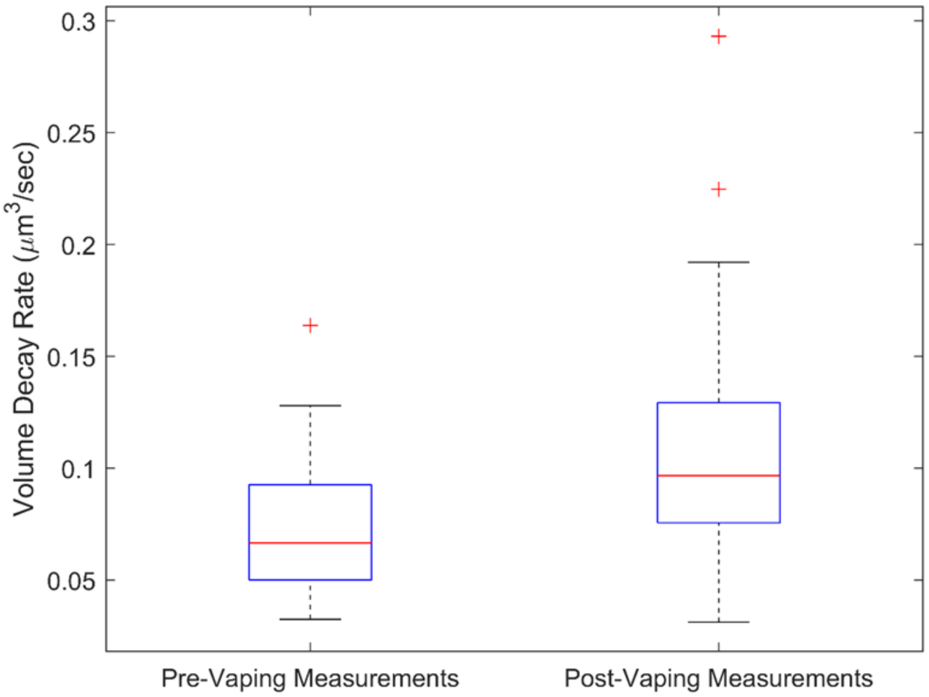

Supplementary Figure S2. Statistical analysis comparing the volume decay rates of pre-vaping and post-vaping aerosol measurements. A statistically significant increase in the volume decay rate of post-vaping aerosol measurements was confirmed by conducting a Wilcoxon rank-sum test ($p<0.05$), which tests the null hypothesis that the volume decay rates are sampled from continuous distributions with equal medians. The central red line on each box indicates the median of the measured volume decay rates, while the bottom and top edges of each box refer to the 25th and 75th percentiles, respectively. The black whisker covers a range of $\mu \pm 2.7\sigma$, where μ is the mean and σ is the standard deviation of the measurement data. Points beyond this range are considered outliers and marked with red + points.

Captions for Supplementary Videos 1-4

Supplementary Video 1: Example movie of a volatile particle. The video is formed using time-lapse microscopic images that are reconstructed from holograms of a volatile particle imaged by c-Air device. The phase channel of the reconstructed microscopic video is displayed in the upper right panel. The estimated volume decay curve of the volatile particle is shown in the upper left panel, with a blue dot pointing to the time spot shown in the corresponding image. The lower right panel shows the amplitude channel of the images, while the lower left panel shows the active pixel count of the particle. The active pixel count, A(t), is calculated from the amplitude channel of the reconstructed images of the particle and it refers to the number of the pixels in the amplitude channel that are one standard deviation greater than the mean value of each image. The red dot points to the time spot shown in the corresponding time-lapse image.

Supplementary Video 2: Example movie of a single semi-volatile particle.

Supplementary Video 3: Example movie of a coagulated semi-volatile particle.

Supplementary Video 4: Example movie of a non-volatile particle.
